# Glutamate Receptor Subunit and Calmodulin Kinase II Expression, with and without T Maze Training, in the Rat Hippocampus following Bilateral Vestibular Deafferentation

**DOI:** 10.1371/journal.pone.0054527

**Published:** 2013-02-07

**Authors:** Yiwen Zheng, Georgina Wilson, Lucy Stiles, Paul F. Smith

**Affiliations:** Department of Pharmacology and Toxicology, School of Medical Sciences, and the Brain Health Research Centre, University of Otago Medical School, Dunedin, New Zealand; University of Pittsburgh, United States of America

## Abstract

Many previous studies have shown that lesions of the peripheral vestibular system result in spatial memory deficits and electrophysiological dysfunction in the hippocampus. Given the importance of glutamate as a neurotransmitter in the hippocampus, it was predicted that bilateral vestibular deafferentation (BVD) would alter the expression of NMDA and AMPA receptors in this area of the brain.

**Methods:**

The expression of the NR1, NR2A, NR2B, GluR1, GluR2, GluR3 and GluR 4 glutamate receptor subunits, as well as calmodulin kinase IIα (CaMKIIα) and phosphorylated CaMKIIα (pCaMKIIα), was measured in the rat CA1, CA2/3 and dentate gyrus (DG) subregions of the hippocampus, at 24 h, 72 h, 1 week, 1 month and 6 months following BVD, using western blotting. In the 6 month group, half of the animals underwent spatial forced alternating training in a T-maze.

**Results and Discussion:**

For the 24 h, 72 h, 1 week and 1 month data, there was no significant effect of surgery for any hippocampal subregion. However, for the 6 month data set, T maze training had a significant effect independently of surgery. The results of these experiments suggest that BVD is not associated with large changes in glutamate receptor subunit or CaMKIIα expression in the rat hippocampus, at least in terms of both the intra-cytoplasmic and membrane receptor subunits together, that western blotting can measure. However, spatial training-associated increases in glutamate receptor and CaMKIIα expression can be induced in BVD rats with impaired spatial performance. Therefore, the neurophysiological changes underlying BVD-induced spatial learning and memory deficits are more likely to be due to up and down regulation or changes in affinity/efficacy of glutamate receptors at the membrane level than changes in subunit transcription and transduction at the intra-cytoplasmic level.

## Introduction

Numerous behavioural studies in animals have demonstrated that lesions of the peripheral vestibular system lead to spatial memory impairments that persist long after the acute vestibular reflex deficits have partially subsided or ‘compensated’ [1]–[9]. These deficits are most severe when the lesions are bilateral and in this case they appear to be more or less permanent [4], [6], [7]. Clinical studies of human patients with bilateral vestibular loss also indicate that spatial memory is impaired, even 5–10 years following the lesions [10]. Electrophysiological studies in animals suggest that the spatial memory impairment following bilateral vestibular deafferentation (BVD) may be partially attributable to a dysfunction of hippocampal place cells [11], [12] and theta rhythm [9], [13], [14]. MRI studies in humans have shown that bilateral vestibular loss is associated with a bilateral atrophy of the hippocampus [10]; however, no reduction in hippocampal volume has been reported in rats with bilateral vestibular lesions [8], [15] and long-term potentiation (LTP) appears to be intact, at least at the level of resolution of field potential recording *in vivo* [16].

While it is clear that functional changes occur in the hippocampus that might explain spatial memory impairment following bilateral vestibular loss, the neurochemical bases of these changes remain unknown. Relatively few data are available on the neurochemical changes that occur in the hippocampus following BVD, in particular those relating to glutamatergic synaptic transmission that might be important for spatial memory and LTP. Previous studies involving unilateral vestibular deafferentation (UVD) in rats, which elicits a severe imbalance in vestibulo-ocular and vestibulo-spinal reflexes that gradually abates over time, showed that the expression of the NR1 and NR2A subunits of the N-methyl-D-aspartate (NMDA) subtype of glutamate receptor, decreased in the ipsilateral CA2/3 region at 2 weeks post-UVD, while the expression of the NR2A subunit was also reduced in the contralateral CA2/3 region at the same time point [17]. On the other hand, the expression of the NR2A subunit was increased in the CA1 region at 10 hs following UVD [17]. This study did not investigate the α-amino-3-hydroxy-5-methyl-4-isoxazolepropionate (AMPA) receptor subunits, GluR1-GluR4, and the longest post-operative time point was 2 weeks. The only study to date to investigate glutamate receptors in the hippocampus following BVD, measured NMDA receptor density and affinity using receptor autoradiography. In this study, Besnard et al. [8] used a sequential UVD procedure, involving intratympanic sodium arsanilate injections (i.e., one ear, followed several weeks later by the other ear), and observed a significant increase in the NMDA receptor B_max_ and a decrease in K_d_ in the hippocampus. This sequential UVD procedure has the advantage of relevance to paroxysmal vestibular disorders in humans in which the right vestibular labyrinth malfunctions, and then the left, or vice versa, e.g. some types of Meniere’s disease [8].

The aim of the present study was to investigate the expression of several glutamate receptor subunits and calmodulin kinase IIα (CaMKIIα) in the CA1, CA2/3 and dentate gyrus (DG) subregions of the hippocampus, at various time points following BVD, using western blotting. For the NMDA receptor, the NR1 subunit was analysed because it is necessary for NMDA receptor function, binding the co-agonist, glycine, while the NR2 subunit binds glutamate [18]. The NR2A and NR2B subunits were measured because they have an important impact on the receptor’s channel conductance, ligand affinity and sensitivity to Mg^2+^ [19]–[22]. For the AMPA receptor, all 4 GluR subunits were measured, GluR1 and GluR2 being the most commonly expressed in the hippocampus, with lower levels of GluR3 and GluR4 [23]–[25]. There is a close relationship between CaMKII and NMDA and AMPA receptor subunits. CaMKII binds to the NR1 and NR2B subunits, and phosphorylates AMPA receptors, thereby altering their channel conductance [26], [27]. Furthermore, activation of NMDA receptors increases the activation of CaMKII, leading to autophosphorylation [28]. Therefore, we also measured CaMKIIα and phosphorylated CaMKIIα (pCaMKIIα) expression in the same hippocampal subregions.

## Materials and Methods

### Animals

At the beginning of the study, male Wistar rats (300–500 g) were randomly allocated to BVD or sham surgery conditions at one of 5 time points: 24 h, 72 h, 1 week, 1 or 6 months post-surgery (n = 7 in each BVD and sham group, for the 24 h, 72 h and 1 week time points; n = 7 for the BVD group and 6 for the sham group for the 1 month time point; and n = 14 for the BVD group and 12 for the sham group at the 6 month time point, making a total of 81 animals). For the 6 month time point, BVD or sham animals were divided into those with or without spatial forced alternation in T maze training (n = 7 or 6 for each group, respectively), to determine whether spatial learning experience had any effect on hippocampal glutamate receptor expression. The animals in this group have previously been reported to exhibit spatial memory deficits [5]. Animals were maintained on a 12∶12 h light:dark cycle at 22°C and housed in individual cages.

### Ethics Statement

All procedures were carried out in accordance with the regulations of the University of Otago Committee on Ethics in the Care and Use of Laboratory Animals and were approved by that Committee.

### Surgery

The animals were anaesthetized using 300 mg/kg fentanyl citrate (i.p.) and 300 mg/kg medetomidine hydrochloride (i.p.) and BVD surgery was performed under microscopic control as detailed previously [4]. Briefly, a retro-auricular approach was used to expose the tympanic bulla. Once exposed, the malleus and incus were removed, the stapedial artery was cauterized; and the horizontal and anterior semicircular canal ampullae and the saccule and utricle were drilled open and their contents aspirated. The sham surgery involved exposing the temporal bone and removing the tympanic membrane without producing a vestibular lesion. Finally, the temporal bone was sealed using dental cement. After the surgical margins had been sutured, a postoperative analgesic, carprofen (5 mg/kg, s.c.), was administered. Previous studies have confirmed, using temporal bone histology, that this BVD procedure produces a complete and permanent bilateral lesion of the vestibular labyrinths with no damage beyond the temporal bone [4].

Following surgery, the animals recovered for 24 h, 72 h, 1 week, 1 month or 6 months. All of the animals receiving a BVD exhibited the postural and locomotor behavioural symptoms that are characteristic of bilateral vestibular loss, although these were not measured quantitatively (see Results). By 1 month the animals had recovered from the severe, acute symptoms of BVD and some compensation had occurred, although the vestibulo-ocular and vestibulo-spinal reflexes never return to normal following BVD [29]. Approximately half of the animals in the 6 month group received training in a T maze, and their performance in this task was quantified and is reported in Zheng et al. [5].

### T Maze Training

The T-maze was modified from a cross maze by blocking one of the arms with a barrier at the entrance of the arm and the rest of the three arms formed a T-shaped maze. Each arm was 50 cm long and 12 cm wide with a sunken food well (2 cm in diameter and 0.75 cm deep) located at the end of the arm. The walls of the arms were 20 cm high. The stem was of the same length, width, and height and a starting area was created by inserting a plastic door 25 cm from the end of the stem. The apparatus was elevated 90 cm above the ground. The rats all had one day of habituation (10 min) to the maze with the food wells in all four arms baited. During this period, the sucrose tablets (Noyes sucrose reward tablet, 45 mg, Research Diet) were continuously replaced so that no arm was found to be empty when first visited. The training was performed according to the procedure described by Zheng et al. [5]. Basically, each trial consisted of two stages. On the first stage (also called the sample run), a barrier was placed at the neck of the T-maze to close off one arm and three sucrose tablets were placed in each food well. The sample run was started by placing the animal in the starting area and removing the barrier. The animal was forced to enter a preselected arm and allowed to eat food there. Then, the animal was picked up and placed in the starting area for a delay of 10 sec (or no delay for sessions 11–20), during which the barrier was removed and the maze was wiped clean. On the second stage (the choice run), once the door was opened, the animal was allowed a free choice between the two arms of the T-maze. If the animal entered the arm not visited previously on the sample run, it received a reward (allowed to eat the food there). If the animal entered the arm visited previously, it was confined to that arm for about 10 sec and then returned to its cage. The criterion for selecting an arm was that the rat placed a hind foot in one of the arms. The animals received eight trials per session for a total of 20 sessions. For sessions 1–10, the animals were given a delay of 10 sec between the sample run and the choice run, while for sessions 11–20, the animals had choice runs straight after the sample runs with no delays. Six animals at a time were carried into the experimental room in an enclosed carry box with six individual compartments. Each of the six rats had one trial in turn so that the inter-trial interval was 3–5 min. An equal number of forced right or left turns was given in a pseudorandom sequence. The number of correct choices was recorded for each session.

In order to control for handling and maze exposure-related stress, two identical T-mazes were placed side by side in the same room. One rat that received the T maze training described above and one rat that did not, were placed in the respective T-mazes at the same time, except that the T-maze-trained rat performed the task while the no-T-maze-trained rat was allowed to freely explore the T-maze for the same duration.

### Tissue Preparation

At the designated time point post-op., the animals were decapitated without anaesthesia, and the hippocampal subregions (CA1, CA2/3 and the dentate gyrus (DG)), were dissected out using the methods described previously [17], [30], and stored in a −80°C freezer until use. The 6 month post-op. rats were sacrificed at 24 h after the last behavioural test and the different groups were counter-balanced for the order of sacrifice in order to control for potential post-training time effects.

At the time of processing, tissue buffer (containing Complete Proteinase Inhibitor, 50 mM Tris–HCl pH 7.6) was added to the samples on ice, then the tissue was homogenised using ultrasonification (Sonifier cell disrupter B-30, Branson Sonic Power Co.) and centrifuged at 12,000 g for 10 min at 4°C. The protein concentration in the supernatant was measured using the Bradford method and equalized, then the supernatants were mixed with gel loading buffer (50 mM Tris-HCl, 10% SDS, 10% glycerol, 10% 2- mercaptoethanol, 2 mg/ml bromophenol blue) in a ratio of 1∶1 and boiled for 5 min.

### Western Blotting

Ten µg of protein from each sample was loaded in each well on a 7.5% SDS-polyacrylamide mini-gel and pre-stained protein markers (10–250 kDa; Bio-Rad, Precision Plus: Dual colour) were used as molecular weight markers on each gel. In order to control for between gel variations, an internal standard made of pooled cerebellar samples from sham rats was loaded on each gel. The samples were electrophoresed with a 90 V variable current (Bio-Rad, PowerPack 3000) until protein flattened at the stacking/resolving interface, and 180 V thereafter. The proteins were transferred to polyvinylidene-difluoride (PVDF) membranes using a transblotting apparatus (2.5 L; Bio-Rad). The transfer was performed overnight in transfer buffer (25% methanol, 1.5% glycine and 0.3% Tris-base) at a 10 V variable current (Bio-Rad PowerPack 3000). Non-specific IgG binding was blocked by incubation with 5% dried milk protein (Pams) and 0.1% bovine serum albumin (BSA) (Sigma) for 6–7 h at 4°C. The membranes were then incubated with affinity-purified polyclonal goat antibodies raised against GluR1, GluR2, GluR3 and GluR 4, and affinity-purified polyclonal rabbit antibodies raised against NMDA ε1 (NR1), NMDA ε2 (NR2A), NMDA ζ1 (NR2B), CaMKIIα and pCaMKIIα, overnight at 4°C (see antibody details in Table 1). The specificity of these antibodies has been demonstrated in previous studies (NR1 [31]; NR2A [32]; NR2B [33]; GluR1 [34]; GluR2 [35]; GluR3 [36]; GluR4 [37]; CaMKIIα [38]; pCaMKIIα [39]; β-actin [40]) and the dilutions were optimised for the current study. The secondary antibodies were anti-goat IgG linked to horseradish peroxidase and anti-rabbit IgG linked to horseradish peroxidase (see details in Table 1). Detection was performed using the enhanced chemiluminescence (ECL) system (Amersham Biosciences, NZ). Hyperfilms (Amersham Biosciences, NZ) were analyzed by densitometry to determine the quantity of protein expressed in each group using a calibrated imaging densitometer (Bio-Rad) and a PowerPC Mac running OS 9.2 and Quantity One software.

**Table 1:** Details of the primary and secondary antibodies used in the western blotting experiments.

Primary Antibody	Dilution	Secondary Antibody	Dilution
GluR1 (C-19, sc-7609), Santa Cruz	1∶1000	donkey-anti-goat IgG, Sigma	1∶5000
GluR2 (N-19, sc-7611), Santa Cruz	1∶1000	donkey-anti-goat IgG, Sigma	1∶5000
GluR3 (N-19, sc-7613), Santa Cruz	1∶1000	donkey-anti-goat IgG, Sigma	1∶5000
GluR4 (C-20, sc-7614), Santa Cruz	1∶1000	donkey-anti-goat IgG, Sigma	1∶5000
NR1 (H-54, sc-9056), Santa Cruz	1∶1000	goat-anti-rabbit, Sigma	1∶1000
NR2A (H-50, sc-9057), Santa Cruz	1∶1000	goat-anti-rabbit, Sigma	1∶1000
NR2B (H-300, sc-9058), Santa Cruz	1∶1000	goat-anti-rabbit, Sigma	1∶1000
CaMKII (H-300, sc-13082), Santa Cruz	1∶1000	goat-anti-rabbit, Sigma	1∶1000
p-CaMKIIα (Thr 286, sc-12886-R), Santa Cruz	1∶1000	goat-anti-rabbit, Sigma	1∶1000
β-actin (I-19, sc-1616), Santa Cruz	1∶5000	donkey-anti-goat IgG, Sigma	1∶5000

Results were expressed as the volume of the band, i.e., optical density × area of the band. An antibody against β-actin (see details in Table 1) was used as a loading control and exploratory regression analyses performed in our laboratory have shown that any changes in β-actin expression were unlikely to account for changes in the target protein expression (R^2^ = 0.087) [30]. The volume of each target band was then normalised to its corresponding loading control and then the internal standard within each gel. It was expected that the protein levels measured would reflect both the intra-cytoplasmic and membrane receptor subunits together.

### Statistical Analysis

The data were tested for normality and homogeneity of variance. If necessary, they were natural log transformed and then re-tested. A series of 1 or 2-way multivariate analyses of variance (MANOVAs), using surgery and time point (24 h, 72 h and 1 week data), surgery (1 month data) or surgery and training (6 month data) as factors, were performed in SPSS 20 for each individual hippocampal region, with the 8 proteins as dependent variables [41], [42]. Pillai’s Trace statistic was used because it has been reported to be more robust against violation of assumptions than other MANOVA statistics [41]. Since the tissue processing for the early time points (24 h, 72 h and 1 week), the middle time point (1 month) and the late time point (6 months) was done at different times, the data from these 3 conditions were analysed using separate MANOVAs, followed by univariate ANOVAs in the case of a significant MANOVA. For the 1 and 6 month conditions, GluR4 and NR2A were not analysed. In order to further investigate the data, and determine whether combinations of variables were changing in addition to individual variables, we performed a series of further multivariate statistical analyses. Linear discriminant analysis (LDA) was performed, with Wilks’ λ as the test statistic and leave one out cross-validation [41], [43]. Finally, cluster analyses were performed on the data expressed as z scores using Ward’s minimal variance algorithm and the correlation coefficient distance [41], [42]. The significance level was set at 0.05 for all comparisons.

## Results

BVD resulted in a number of postural and locomotor behavioural symptoms that are characteristic of bilateral vestibular loss. These included: gait ataxia, marked hyperactivity, head-dorsiflexion, head-weaving, and circling [3]–[9], [44]. In no case were these behaviours seen in the sham-operated animals. In addition, the BVD animals in the 6 month condition were demonstrated to have significant memory impairment in the spatial forced alternation T maze task, as reported in Zheng et al. [5].

### Protein Expression in the Hippocampus at 24 Hours, 72 Hours and 1 Week post-BVD

**Figure 1:**
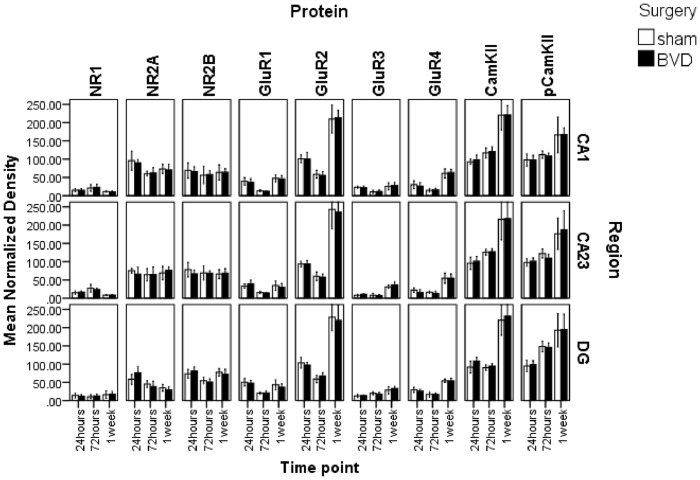
Mean normalized density of expression of NR1, NR2A, NR2B, GluR1, GluR2, GluR3, GluR4, CaMKIIα and pCaMKIIα in the CA1, CA2/3 and DG regions of the hippocampus at 24 h, 72 h and 1 week following BVD or sham surgery. Error bars represent 95% confidence intervals for the mean.

There was no significant effect of surgery on the protein expression for any hippocampal subregion, and no significant interaction between surgery and time point, for the 24 h, 72 h and 1 week conditions. However, there was a significant effect of time point on the protein expression in all cases (CA1: F(18,58) = 29.87, P≤0.000; CA2/3: F(18,58) = 35.38, P≤0.000; DG: F(18,58) = 57.30, P≤0.000; Fig. 1). Using a LDA on the CA1, CA2/3 or DG data, no linear discriminant function could be identified that significantly predicted whether the brain tissue came from a BVD or a sham animal.

### Protein Expression in the Hippocampus at 1 Month Post-BVD

The results were similar for the 1 month data set: there were no significant differences between BVD or sham animals either in the MANOVA or univariate ANOVAs for any hippocampal subregion (data not shown). Using a LDA, no linear discriminant function could be identified that significantly predicted whether the brain tissue came from a BVD or a sham animal.

### Protein Expression in the Hippocampus at 6 Months Post-BVD

**Figure 2:**
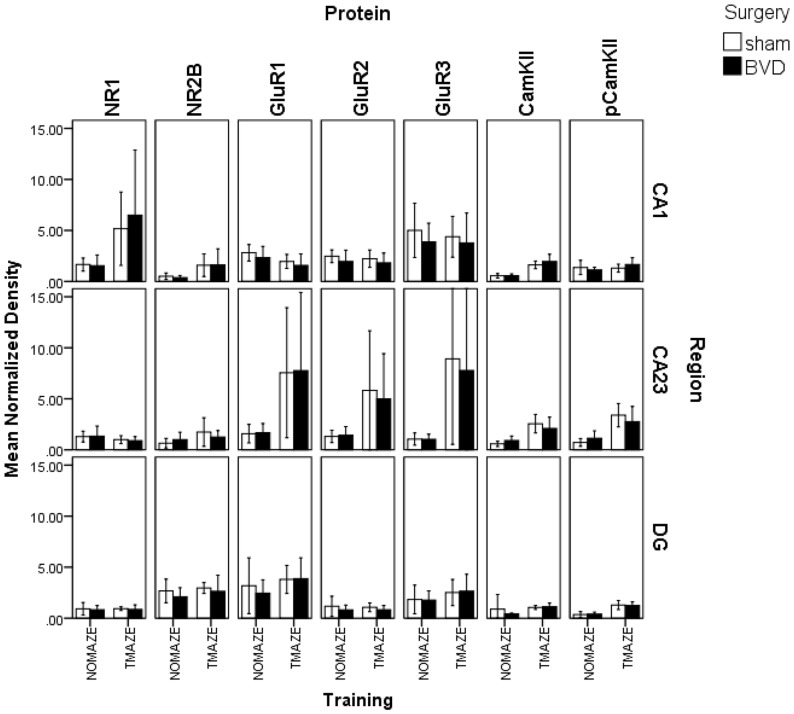
Mean normalized density of expression of NR1, NR2B, GluR1, GluR2, GluR3, CaMKIIα and pCaMKIIα in the CA1, CA2/3 and DG regions of the hippocampus at 6 months following BVD or sham surgery for animals trained in a T maze or not trained in a T maze. Error bars represent 95% confidence intervals for the mean.

The results for the 6 month data set were more complicated. For the MANOVA for CA1, surgery was still non-significant, as was the interaction between surgery and training. However, T maze training had a significant effect on the protein expression (F(7,13) = 16.60, P≤0.000). Univariate ANOVAs indicated that spatial training significantly increased protein expression for CaMKIIα (F(1,19) = 43.22, P≤0.000), NR1 (F(1,19) = 8.02, P≤0.01) and NR2B (F(1,19) = 7.89, P≤0.01), whereas GluR1 (F(1,19) = 5.45, P≤0.03) significantly decreased in both sham and BVD animals (Fig. 2). There were no significant surgery and training interactions in the univariate ANOVAs.

**Figure 3:**
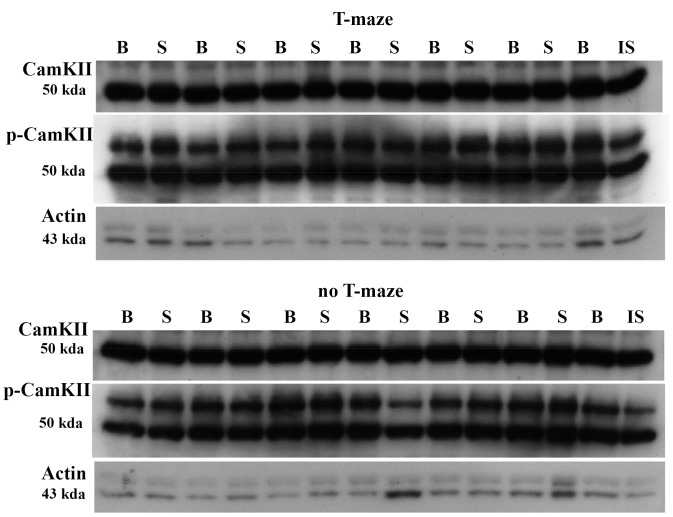
Example of western blots for CamKIIα and pCaMKIIα in CA2/3 for the BVD (‘B’) and sham (‘S’) animals that received T maze training or no T maze training at 6 months post-op. ‘IS’ is the internal standard and β-actin (‘Actin’) is also shown.

For the MANOVA for CA2/3, surgery and the interaction between surgery and training were again non-significant. However, training was significant (F(7,9) = 4.92, P≤0.02), with significant univariate ANOVAs for CaMKIIα (F(1,15) = 20.04, P≤0.000), pCaMKIIα (F(1,15) = 21.83, P≤0.000), GluR1 (F(1,15) = 15.31, P≤0.001), GluR 2 (F(1,15) = 20.64, P≤0.000) and GluR3 (F(1,15) = 17.55, P≤0.001; Figs. 2 and 3). There were no significant interactions in the univariate ANOVAs.

The results were similar for the DG: the MANOVA was significant only for training (F(7,12) = 6.15, P≤0.003), with significant univariate ANOVAs only for CaMKIIα (F(1,18) = 47.29, P≤0.000), pCaMKIIα (F(1,18) = 33.73, P≤0.000), GluR1 (F(1,18) = 9.68, P≤0.006) and GluR3 (F(1,18) = 5.88, P≤0.03; Fig. 2). There were no significant interactions in the univariate ANOVAs.

**Figure 4:**
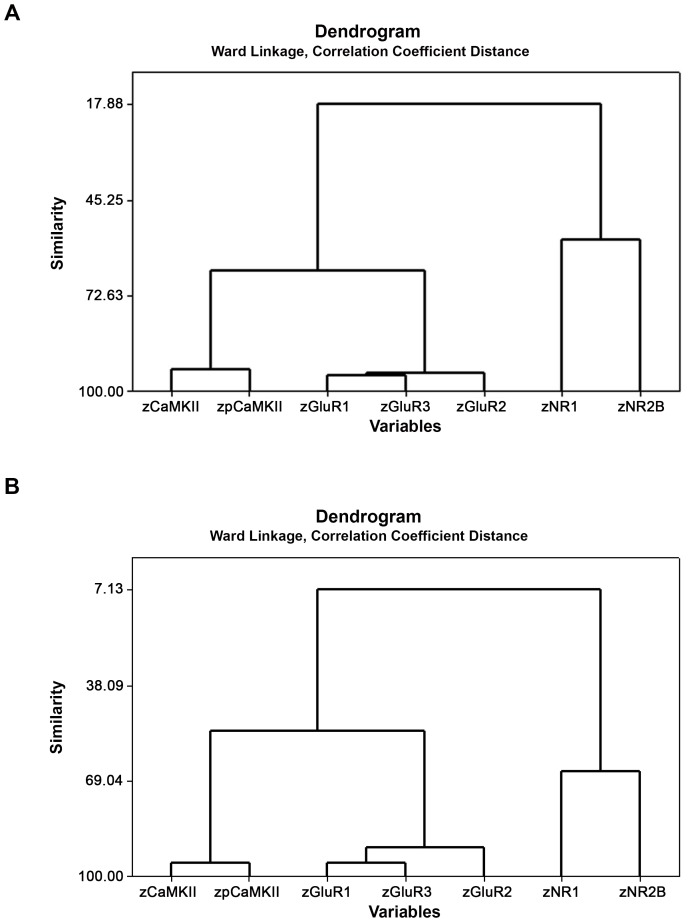
Cluster analysis, using the correlation coefficient distance and Ward’s minimal variance algorithm, on the neurochemical data at 6 months post-op. for the CA1, CA2/3 and the DG data together, showing the relationship between the different neurochemical variables as z scores (zCaMKII etc) for the sham (A) and BVD (B) animals. Note that there is no difference between the clusters for the sham and BVD animals.

**Figure 5:**
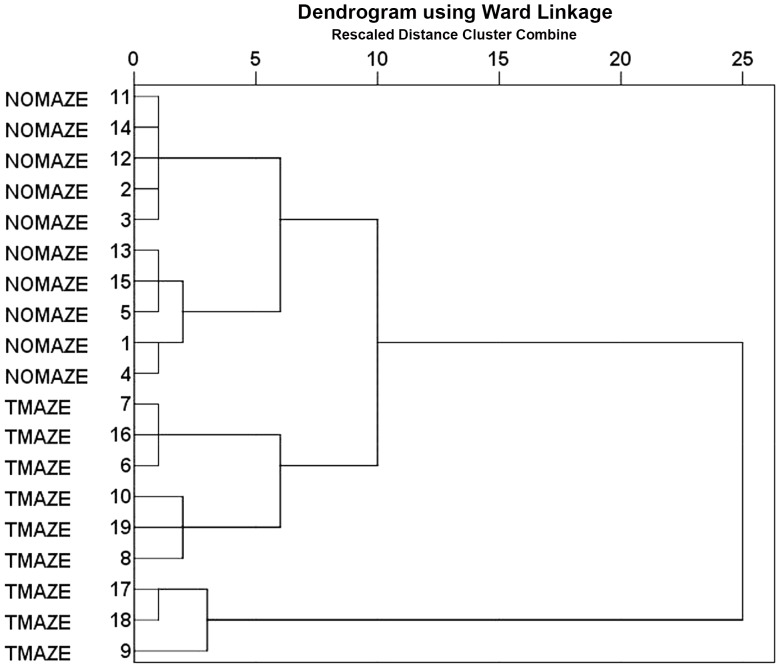
Cluster analysis, using the correlation coefficient distance and Ward’s minimal variance algorithm, on the neurochemical data at 6 months post-op. in CA2/3, showing individual animals according to training (T maze or no T maze). Note that the neurochemical data for CA2/3 can completely distinguish the trained from the non-trained animals.

Using a LDA on the CA1, CA2/3 or DG data, no linear discriminant function could be identified that significantly predicted whether the brain tissue came from a BVD or a sham animal. Cluster analyses also confirmed that surgical group could not be predicted from the neurochemical variables (see Fig. 4 for a comparison of the sham and BVD groups). Training could not be predicted from the neurochemical variables for CA1 and the DG; however, a cluster analysis of the CA2/3 data revealed that the maze and non-T maze-trained animals could be accurately separated based on the neurochemical variables (Fig. 5).

## Discussion

The results of this study show that, using western blotting, the expression of AMPA and NMDA glutamate receptor subunits, and CaMKIIα, in the hippocampus is not significantly different in BVD compared to sham animals at 24 h, 72 h, 1 week, 1 month or 6 months post-op., at least in terms of the intra-cytoplasmic and membrane receptor subunits together. Spatial training in a T maze, however, had a significant effect on the expression of CaMKIIα, NR1, NR2B and GluR1 in CA1, on CaMKIIα, pCaMKIIα, GluR1, GluR2 and GluR3 in CA2/3, and on CaMKIIα, pCaMKIIα, GluR1, and GluR3 in the DG. However, this effect occurred independently of surgery. The results of the LDAs showed that no linear discriminant function could be found that significantly discriminated the BVD from the sham animals on the basis of the neurochemical data.

In a previous study, we observed a decrease in NR1 expression in the ipsilateral CA2/3 region at 2 weeks following UVD [17]. Besnard et al. [8], who performed sequential UVD’s several weeks apart using intratympanic sodium arsanilate injections, observed a significant up-regulation of NMDA receptors in the hippocampus, with reduced affinity, using receptor autoradiography. These findings appear to be in disagreement with our current results. However, there are several differences between the studies that probably account for the apparent discrepancy. First and most importantly, UVD results in an imbalance in the vestibulo-ocular (VOR) and vestibulo-spinal reflexes (VSR), causing symptoms such as spontaneous ocular nystagmus (SN, with quick phase toward the intact side) and postural asymmetry toward the lesioned side (see [29] for a review). These symptoms, which are a result of an imbalance between the left and right central vestibular systems, are so severe initially, that animals such as rats and guinea pigs have difficulty standing immediately after recovery from anaesthesia. Gradually, over a period of 2–3 days, the SN and postural asymmetry decrease in severity in a process known as ‘vestibular compensation’ (see [29] for a review). If a UVD is then performed on the contralateral side after compensation has occurred for the first UVD, this generates SN and postural asymmetry in the opposite direction to the original symptoms, in a phenomenon known as Bechterew’s syndrome (see [29] for a review). Following BVD, in which one labyrinth is lesioned after the other under anaesthesia, SN and postural asymmetry do not occur, because there is no imbalance in activity between the two labyrinths following recovery from the anaesthetic. Rather, BVD results in a complete loss of the VORs and VSRs. Therefore, the behavioural symptoms which follow UVD or two UVD procedures in sequence, are quite different from those that follow a simultaneous BVD under anaesthesia. The most likely explanation for the difference between our results for the NR1, NR2A and NR2B subunits of the NMDA receptor and Besnard et al.’s [8] results for the NMDA receptor, is the different temporal sequence of the lesions. However, another important difference is that Besnard et al. [8] used Sprague Dawley rats, whereas we used Wistar rats. It must also be considered that whereas we used surgical lesions of the labyrinth, Besnard et al. [8] used intratympanic injections of the ototoxin, sodium arsanilate. The sodium arsanilate method has been demonstrated to destroy the vestibular hair cells in the vestibular labyrinth without damaging the VIIIth nerve dendrites, axons or primary afferent neurons in Scarpa’s ganglion [45]. By contrast, surgical lesions of the rat labyrinth have been reported destroy the vestibular hair cells as well as damage some VIIIth nerve dendrites [46]. Both models induce severe spatial memory deficits that should lead to similar consequences in terms of receptor changes. However, we do not know if remaining ectopic vestibular inputs might be generated by the vestibular system via the intact vestibular nerve with the chemical model, therefore explaining the long-term difference in glutamate receptor expression observed in the two models. Besnard et al. [8] also conducted their analyses of the whole hippocampus at 2 months after the second lesion, whereas we analysed 3 separate hippocampal subregions at 24 h, 72 h, 1 week, 1 month and 6 months post-BVD. Finally, we used western blotting to analyse NMDA receptor subunit expression, whereas Besnard et al. [8] used receptor autoradiography to measure the NMDA receptor number and affinity. Whereas quantitative receptor autoradiography, using beta-imaging, allows for quantification of membrane receptor density, i.e. functional receptors, with a resolution of approximately 150 to 200 µm, western blotting is a semi-quantitative method that quantifies both intra-cytoplasmic and membrane receptor subunits together [47], [48]. One possibility is that the increase in hippocampal NMDA receptor expression observed by Besnard et al. [8] was a response to the sequence of UVD behavioural syndromes. However, this seems too simplistic an explanation, since we did also observe a decrease in NR1 expression in the ipsilateral CA2/3 region, using western blotting, at 2 weeks following UVD [17]. It is possible that the up-regulation occurs in response to the change in vestibular input to the hippocampus as the second UVD occurs. Whatever the explanation, since both sequential and simultaneous vestibular lesions occur clinically in humans, both paradigms are of interest in terms of their effects on spatial memory and the hippocampus.

It was very surprising to find no significant change in the expression of the different NMDA and AMPA receptor subunits, and CaMKIIα, in the hippocampal subregions following BVD. Given the evidence that hippocampal place cell firing and theta rhythm are dysfunctional following BVD [9], [11]–[14] and hippocampal field potentials are reduced in hippocampal slices from rats that had received a UVD several months previously [49], we predicted changes in glutamate receptor subunit expression. One possibility is that the receptor changes that underlie the physiological abnormalities in the hippocampus that are caused by BVD, are too subtle to be detected using western blotting, that they are membrane-specific, and can only be detected using receptor autoradiography with beta-imaging [8]. However, it is conceivable that many of the neurophysiological changes that take place in the hippocampus following BVD do not require changes in the expression of glutamate receptors, but occur as a result of changes in receptor affinity or efficacy, or perhaps do not require receptor plasticity at all. Interestingly, when we analysed field potentials in anaesthetised or alert behaving animals following BVD, we found no significant differences in baseline field potentials or in the induction or maintenance of long-term potentiation [16].

It could be argued that the lack of a significant difference between sham and BVD animals was merely due to experimental error. However, we did find significant effects of T maze training in all hippocampal subregions at 6 months post-op., and these effects were usually an increase in the expression of glutamate receptor subunits, as well as CaMKIIα and pCaMKIIα. In CA1, CaMKIIα, NR1 and NR2B expression were significantly increased, and the expression of GluR1 was significantly decreased. In CA2/3, CaMKIIα and pCaMKIIα expression were significantly increased, as was the expression of GluR1-3. In the DG, CaMKIIα and pCaMKIIα expression were also significantly increased, as was the expression of GluR1 and GluR3. These results are consistent with previous studies in showing that experience can alter the expression of glutamate receptor subunits in the hippocampus (e.g., [50], [51]). For example, Ghafari et al. [50] found that C57BL/6J mice that were trained in a multiple T maze, exhibited a significant increase in the expression of GluR1 and a significant decrease in the expression of GluR2, in the hippocampus. It was particularly interesting that, using cluster analysis in the current study, the expression of the neurochemical variables in CA2/3 could reliably distinguish between the animals that received T maze training and those that did not. These results also demonstrate that significant changes in protein expression could be detected using our assays, and that the lack of effect of BVD was unlikely to be due to methodological problems.

It was surprising to see that spatial training resulted in an increased protein expression of glutamate receptors and CaMKIIα in the hippocampus in the same BVD rats that were impaired in spatial alternation [5]. It has been shown that performance in T-maze spatial alternation is impaired by the NMDA receptor antagonist, D-(-)-2-Amino-5-phosphonopentanoic acid (D-AP5) and in GluR1 knockout mice [52], [53], which suggests that NMDA and AMPA receptors are important for spatial alternation. However, in the present study, spatial training produced the same degree of increase in protein expression in both sham and BVD rats when compared to the untrained rats, regardless of their spatial alternation performance. This, together with our previous finding that LTP is intact in BVD rats [16], suggests that learning and memory impairment in BVD animals cannot be explained simply by altered glutamate receptor plasticity. On the other hand, it must be remembered that rats with BVD have no VOR function, poor VSR function and an altered cognitive representation of both verticality and 3 dimensional space; it is not clear what the neurochemical effects of these deficits might be in the hippocampus.

Overall, the results of these experiments suggest that BVD is not associated with large changes in glutamate receptor subunit or CaMKIIα expression in the rat hippocampus, at least in terms of both the intra-cytoplasmic and membrane receptor subunits measured together, but that the neurophysiological changes that occur are more likely to be due to smaller, more subtle alterations in membrane receptor subunits, or in receptor affinity and/or efficacy.
